# A Novel Device for the Evaluation of In Vitro Bacterial Colonization in Membranes for Guided Tissue and Bone Regeneration

**DOI:** 10.3390/dj12070202

**Published:** 2024-06-29

**Authors:** Ana Clara Kuerten Gil, Eugenio A. D. Merino, Diogo Pontes Costa, César Nunes Giracca, Ricardo Mazzon, Gabriel Leonardo Magrin, Josiane de Almeida, Cesar Augusto Magalhães Benfatti

**Affiliations:** 1 Department of Implant Dentistry, Federal University of Santa Catarina, Florianópolis 88040-900, Brazil; anaclarakuertengil@gmail.com (A.C.K.G.); cesarambenfatti@gmail.com (C.A.M.B.); 2 Department of Production Engineering, Federal University of Santa Catarina, Florianópolis 88040-900, Brazil; eugenio.merino@ufsc.br (E.A.D.M.); diogopontes102@gmail.com (D.P.C.); eng.giracca@gmail.com (C.N.G.); 3 Department of Microbiology, Federal University of Santa Catarina, Florianópolis 88040-900, Brazil; ricardo.mazzon@ufsc.br; 4 Department of Endodontics, University of South Santa Catarina, Florianópolis 88010-010, Brazil; dealmeidajosiane@hotmail.com

**Keywords:** bacteria, 3D, CAD/CAM, guided tissue regeneration, guided bone regeneration

## Abstract

**Purpose:** To evaluate, in vitro, the efficiency of a novel apparatus to test the adherence and penetration of bacteria on different membranes for guided regeneration. **Methodology:** To create the 3D device, Computer Aided Design/Computer Aided Manufacturing (CAD/CAM) systems were used. Three types of biomaterials were tested (*n* = 6): (DT) a collagen membrane; (DS) a polymer membrane; and (LP) a dense polytetrafluoroethylene barrier. The biomaterials were adapted to the apparatuses and challenged with two different monospecies bacterial culture of *A. actinomycetemcomitans b* and *S. mutans*. After 2 h, bacterial adherence and penetration were quantified by counting the number of colony-forming units (CFUs). Two specimens from each group were used for image analysis using Confocal Laser Scanning Microscopy. Statistical analysis was performed. **Findings:** The DS group had a higher adherence of *S. mutans* compared to *A. actinomycetemcomitans b* (*p* = 0.05). There was less adherence of *A. actinomycetemcomitans b* in the DS group, compared to the LP (*p* = 0.011) and DT (*p* < 0.001) groups. Only the membranes allowed penetration, which was blocked by barriers. The DT group allowed a greater penetration of *S. mutans* to occur compared to *A. actinomycetemcomitans b* (*p* = 0.009), which showed a higher penetration into the DS membranes compared to *S. mutans* (*p* = 0.016). The penetration of *A. actinomycetemcomitans b* through DS was higher compared to its penetration through DT and LP (*p* < 0.01 for both). DT and DS allowed a greater penetration of *S. mutans* to occur compared to LP, which prevented both bacterial species from penetrating. **Conclusion:** The apparatus allowed for the settlement and complete sealing of the biomaterials, enabling standardization.

## 1. Introduction

In guided tissue (GTR) and bone regeneration (GBR) surgeries, membranes serve a crucial function by guiding and promoting the regeneration of specific tissues while preventing the ingress of unwanted cell types [1]. These membranes create a secluded space that allows for the selective repopulation of cells, favoring the growth of desired tissues such as bone or periodontal ligaments [2]. There are several types of biomaterials that are commercially available, from resorbable to non-resorbable membranes and barriers [3]. The utilization of non-resorbable membranes necessitates secondary surgeries for membrane removal, commonly exhibiting the occurrence of flap sloughing and exposure, which predisposes patients to infections and unfavorable outcomes [4]. To address these limitations, absorbable barriers like collagen membranes have been developed as alternatives [4,5].

Bacterial contamination of membranes and barriers exposed to the oral environment has been shown to be linked to the failure of periodontal and bone regeneration treatments [6]. This exposure may culminate in the early removal of these biomaterials, whose maintenance of space may be compromised, giving rise to the proliferation of undesirable cells at the site to be regenerated [7]. Based on this, the authors strongly endorse the total coverage of membranes with soft tissue, associated with an appropriate suture technique [8].

However, the structural and chemical properties of membranes and barriers seem to be directly related to the bacteria’s ability to adhere to and penetrate through them [9]. Thus, membranes with a higher roughness, such as resorbable collagen membranes, tend to accumulate microorganisms more easily [10]. In contrast, surfaces with fewer irregularities, like dense polytetrafluorethylene (d-PTFE) non-resorbable barriers, have lower propensity [11]. This property allows the latter material to be continually exposed to the oral environment since the likelihood of contamination is decreased, and its structure acts as a barrier, preventing bacterial cells from passing into the operated site [7].

Containing the contamination of biomaterials in the trans and postoperative periods may dictate the outcome of the regenerative process [8]. Evidence shows a greater increase in clinical attachment in guided tissue regeneration (GTR) in cases where there was no membrane exposure, as well as a more favorable prognosis in the osseointegration of implants associated with guided bone regeneration (GBR) [7]. Furthermore, the premature exposure of a membrane can create a reservoir of microorganisms, which can not only infect viable periodontal tissue, but also facilitate the recurrence of periodontitis in individuals with higher levels of periodontal pathogens [12,13].

A wide variety of periodontal pathogens have already been identified in the adhesion and penetration of regenerative biomaterials [14,15,16,17], along with numerous inflammatory cells [18]. The colonization of microorganisms occurs in the first few minutes of exposure and can last for weeks [15]. Species such as *Streptococcus mutans*, a pioneer in the formation of periodontal biofilm, and *Aggregatibacter actinomycetemcomitans*, capable of inhibiting fibroblast synthesis and inducing bone resorption, have already been related to the contamination of biomaterials [16]. The pathogenicity of the periodontal microbiota draws attention to a possible interference in the useful life of biomaterials, such as their degradation due to enzymes and toxins, which directly affects the regenerative process [19,20].

The current literature lacks a standardized method to test the bacterial adherence and penetration characteristics of membranes and barriers. This creates a series of biases that can cause scientific uncertainties when establishing processes in the face of obstacles promoted by microbes in the oral cavity. Also, the dental market is being perfected with each passing day, with new technologies and biomaterial alternatives being used in GTR and GBR, often bringing these products to routine clinical application before reliable laboratory tests. There is a gap in the literature regarding the contamination of co-polymer polylactic acid, glycolic poliacid and politrimethylene carbonate membranes (Duosynt^®^). To our knowledge, this study is the first to test the behavior of this biomaterial in the face of bacterial adherence and penetration.

Considering the lack of a standardized device to assess and compare membrane contamination in laboratory tests, the aim of this research is to evaluate, in vitro, the behavior of different commercially available membranes and barriers, through the use of a novel specific methodological apparatus manufactured using a CAD/CAM system, to test bacterial adherence to and penetration of membranes and barriers used in GTR and GBR.

## 2. Materials and Methods

### 2.1. Bacterial Species and Inoculum Preparation

The bacteria cell lines were obtained from The University of Guarulhos, Brazil, and both species were provided by Prof. Bruno Bueno. All the steps of the bacterial experiments were performed at the Bacterial Molecular Genetics Laboratory (GeMBac) at the Federal University of Santa Catarina (UFSC). The facultative anaerobic bacterial species *Aggregatibacter actinomycetemcomitans b* (ATCC 29523) and *Streptococcus mutans* (ATCC 25175) were used. The microorganisms were collected from freezer stock at −80 °C. A fresh culture of each species was obtained through overnight incubation of 500 µL of the stock in 3 mL of Brain Heart Infusion (BHI) culture medium, pH 7.1, at 37 °C, in aerobiosis. Prior to the experiment, the optical density of each culture was corrected to OD600 ≈ 0.5.

### 2.2. Experimental Groups

Three groups of different biomaterials were formed: (DT) Lumina Coat Double Time^®^ collagen membrane (predominantly type I and III natural bovine collagen) (2 × 20 × 30 mm) (Criteria, San Carlos, SP, Brazil); (DS) Duosynt^®^ polymer membrane (polylactic acid, glycolic poliacid and politrimethylene carbonate) (2 × 20 × 20 mm) (FGM Dental Group, Joinville, SC, Brazil); and (LP) Lumina PTFE^®^ d-PTFE barrier (1 × 20 × 30 mm) (Criteria, San Carlos, SP, Brazil).

### 2.3. Methodological Apparatus

The methodological apparatus used to adapt the tested biomaterials was developed by the Management and Design Center at the Design and Usability Laboratory of the Department of Management, Media and Technology, associated with the post-graduate program of Production Engineering at the Federal University of Santa Catarina, Florianópolis, Brazil.

The design of the apparatus consisted of 4 pieces, which were connected through a threading system to form a specific device for testing bacterial adhesion and penetration in membranes and barriers.

**Figure 1:**
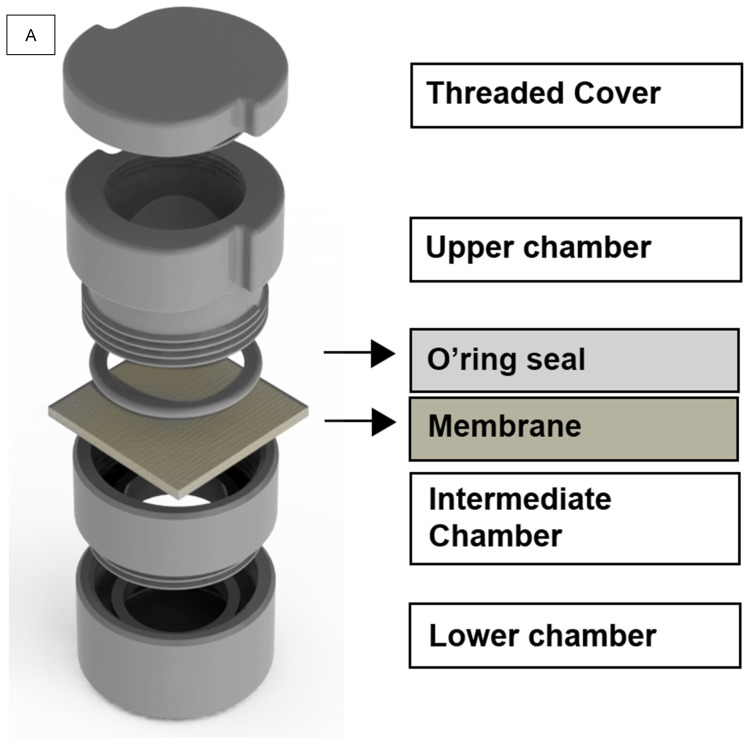

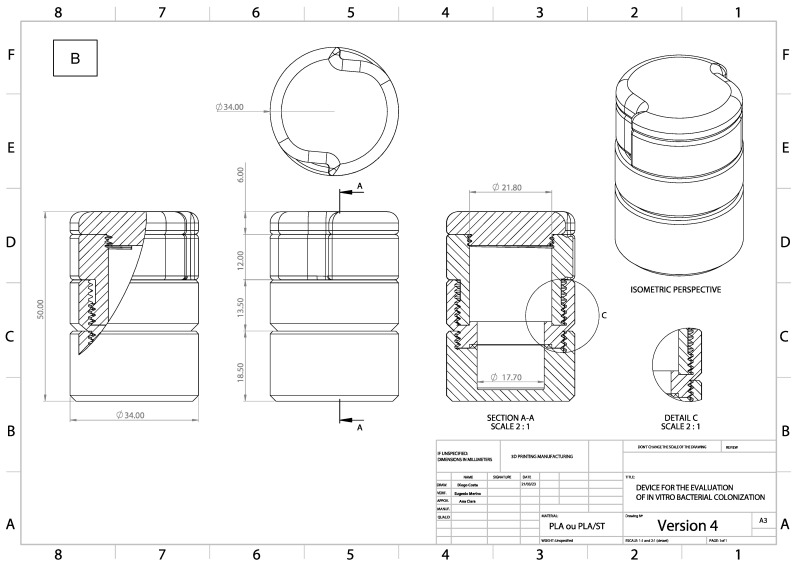
(**A**,**B**). Methodological apparatus. (**A**) Virtual design of the methodological apparatus, with unthreaded chambers (elaborated by the authors). (**B**) Total and individual measurements for each component of the methodological apparatus (elaborated by the authors).

The first piece, or lower chamber (Ø34 mm × 18.5 mm), consisted of a compartment with a closed and an open end, and its purpose was to store the sterile BHI culture medium. The piece that threads into the lower chamber, the intermediate chamber (Ø34 mm × 13.5 mm), was designed to fit the testing biomaterial, in which the upper surface is placed in contact with bacterial inoculum, and the lower surface is in contact with the sterile liquid of the lower chamber. The upper chamber, in turn, is the piece that threads in the intermediate chamber, with the aid of a rubber O’ring (Ø22 mm × 3 mm), whose role comprises the sealing of the apparatus in order to prevent the leakage of bacterial inoculum that dripped on the upper surface of the biomaterial. Finally, the last piece comprises a threaded cover (Ø34 mm × 7.54 mm) to prevent external contamination and possible evaporation of the inoculum liquid (Figure 1A,B).

For the elaboration of the 3D prototype model, the *Onshape* software(version 1.182, PTC Products, Cambridge, MA, USA) from the Computer Aided Design (CAD) system was used. Subsequently, the CAD file generated in Standard Triangle Language (STL) format was customized using the 3DPrinterOS program (3D Control Systems, San Francisco, CA, USA), through the Computer Aided Manufacturing (CAM) system. At this stage, the direction and positioning of printing the apparatus were stipulated to use the material and finish the surface better and preserve the virtually designed geometry. Parameters were defined regarding the printer configuration (Table 1) and printing of the methodological apparatus (Table 2).

The 3DPrinter Prime 1 printer (GTMax 3D, Americana, SP, Brazil) and polymeric filament of modified polylactic acid (PLA/ST) were used for printing. After the device was printed, post-printing care was performed, which consisted of removing traces of material and the structural layer using precision styluses and scalpels. Granulation sandpaper of 120 μM, 220 μM, 400 μM and 600 μM was used to perform the fine finishing. Finally, the parts were sanitized with isopropyl alcohol.

### 2.4. Aseptic Conditions

In order to perform the experiment without external contamination, all tested membranes and barriers were obtained in sterile form. The devices were sterilized using ethylene oxide (Sterilab, Curitiba, PR, Brazil) and the laboratory materials were autoclaved at 121 °C for 20 min prior to the experiments. The installation of the biomaterials on the devices was performed inside a laminar flow chamber over a sterilized fabric surgical field, with the help of a sterile tweezer, to ensure that all steps were carried out without contamination. The investigators were vested with sterile gloves, disposable caps and masks during all the procedure.

### 2.5. Growth of the Bacteria

The lower chambers of the apparatuses were filled with 2.5 mL of sterile BHI. Next, the biomaterials in each group (*n* = 6) were placed, individually, on the intermediate chamber of the devices, maintaining the lower surface of the biomaterial in contact with sterile culture medium and sealing the entire diameter of the apparatus. An aliquot of 100 µL of inoculum of each bacterial species was dropped over the upper surface of the biomaterial, separately (1:100 diluted; final concentration ≈ 10^6^ CFU/mL), through the upper chamber of the devices. The sets were incubated at 37 °C, in an aerobic environment, for 2 h. A Three repeats were performed for this step of the experiment.

### 2.6. Bacterial Adherence Analysis

To assess the number of viable cells adhered to the membranes and barriers, after 2 h, the biomaterials were removed from the apparatus with the help of a sterile tweezer. Then, they were rinsed in 0.9% sterile saline baths (3 × 1 mL) in order to dislodge the non-adherent cells and transferred to tubes containing 1 mL of phosphate-buffered saline (PBS). The biomaterials were then sonicated in an ultrasonic bath for 15 min at 40 kHz to dislodge adherent cells and break bacterial aggregates. The obtained bacterial suspensions were vortexed at a speed of 3200 rpm for 1 min, and 100 µL of the suspensions were plated in BHI agar, in duplicate. The plates were incubated in aerobic environment for 24 h, and the number of Colony Forming Units (CFU/mL) was determined. Three repeats were performed for this step of the experiment.

### 2.7. Bacterial Viability Analysis—Confocal Laser Confocal Scanning Microscopy

The image analysis was performed at the Multi-User Laboratory for Studies in Biology (LAMEB) and Central Electron Microscopy Laboratory (LCME). Adherence of viable and non-viable microorganisms on the tested membranes and barriers was analyzed through Confocal Laser Scanning Microscopy (Olympus Europa Holding GmbH, Hamburg, Germany) (*n* = 1). The specimens were cut according to the confocal analysis standard and positioned with the part exposed to the bacterial inoculum facing a glass base (20 mm in diameter and 0.17 mm thick). To determine bacterial viability, the “Live/Dead” kit (BacLightTM kit L-13152; Molecular Probes, Inc., Eugene, OR, USA) was used. The biofilm was stained with the dyes SYTO 9 and propidium iodide, which was applied to the specimens for 20 min in a 1:1 ratio (total volume = 200 μL) and kept away from light. The microscope was set to 488 nm laser emission for SYTO 9 and 514 nm emission for propidium iodide. The maximum excitation/emission for these dyes are 480/500 nm for SYTO 9 and 490/635 nm for propidium iodide, respectively. Specimens were observed using a 20× magnification oil immersion objective lens (200 (GENERAL) and x4 800) with a numerical aperture of 1.4 and the confocal pinhole set to a diameter of 60 μm. The fluorescence of the stained microorganisms was viewed and the images were processed by the BioImageLTM V software. 2.0 (Developed by Dr. Luis Chávez de Paz) with a resolution of 1024 pixels 3 and a zoom factor of 1.0 (1 and 4), resulting in a final pixel resolution of 0.41 mm/pixel. Thirty-four-micrometer deep scans (1 μm step size, 35 cuts/scan) were obtained from each specimen. Cells stained in green represent viable microbial cells, while those stained in red indicate dead microbial cells. This step of the experiment was performed a single time.

### 2.8. Bacterial Penetration Analysis—Bacterial Quantification through CFU Count

After 2 h, the biomaterials were removed from the apparatuses and the culture medium that was stored in the lower chamber, which was initially sterile, was evaluated. Turbidity indicated penetration of microorganisms, and 100 µL of the permeate was collected from each device and plated in BHI agar for CFU count. Three repeats were performed for this step of the experiment.

### 2.9. Validation of the Apparatus Sealing System

Before the experiments with bacterial inoculation, the methodological apparatus was tested to test the sealing of the set and liquid leakage. For this, the lower chamber of the apparatus was filled with 2.5 mL of sterile BHI, and the same culture medium was also placed over an impermeable barrier of dense polytetrafluoroethylene. The device was kept, capped, at room temperature inside a container. After 48 h, we evaluated whether there was liquid extravasation through the structure of the apparatus, observing if there was culture medium contained in the external container. Three repeats were performed for this step of the experiment.

### 2.10. Statistical Analysis

For biofilm quantification, the CFU/mL mean value of each specimen was determined, and the data were normalized through a log10 transformation of each CFU/mL. The obtained data were analyzed through the two-way ANOVA (MANOVA) multivariate test and post hoc Bonferroni at a 5% significance level. Analysis was performed on SPSS 21.0 software (IBM, Armonk, NY, USA).

## 3. Results

### 3.1. Bacterial Adherence Test

#### CFU Count

**Figure 2:**
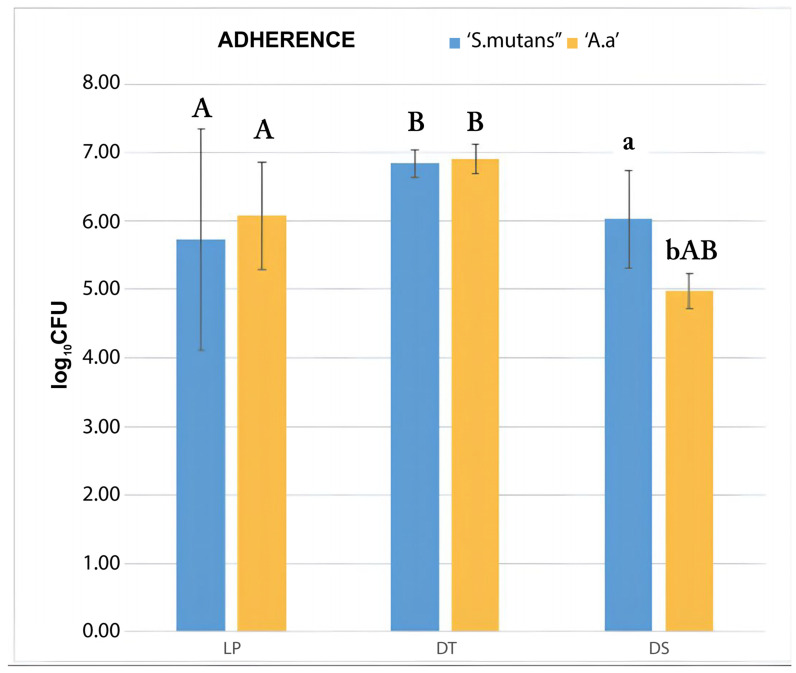
Bacterial adherence test by CFU counting. Average values from the triplicate analysis. Upper case letters indicate statistical difference between experimental groups and lower-case letters indicate statistical difference between bacterial species, in the groups. DS group showed higher adherence of *S. mutans* than *A. actinomycetemcomitans b* and lower adherence of *A. actinomycetemcomitans b* compared to LP and DT groups. DT group showed higher adherence of *S. mutans* compared to LP group, whose adherence was the lowest among the groups (elaborated by the authors).

The experimental group DS allowed a significantly higher amount of adhesion of *S. mutans* than that of *A. actinomycetemcomitans b* (*p* = 0.05). There was also lower amount of adhesion of *A. actinomycetemcomitans b* in the DS group, compared to the amount of adhesion in the LP (*p* = 0.011) and DT (*p* < 0.001) groups. The DT group allowed a significantly higher amount of adhesion of *S. mutans* compared to the LP group (*p* = 0.01), in which the adhesion was the lowest among the tested membranes. The results can be visualized schematically in Table 3 and in the form of a graph in Figure 2

### 3.2. Bacterial Viability (Confocal Laser Scanning Microscopy)

**Figure 3:**
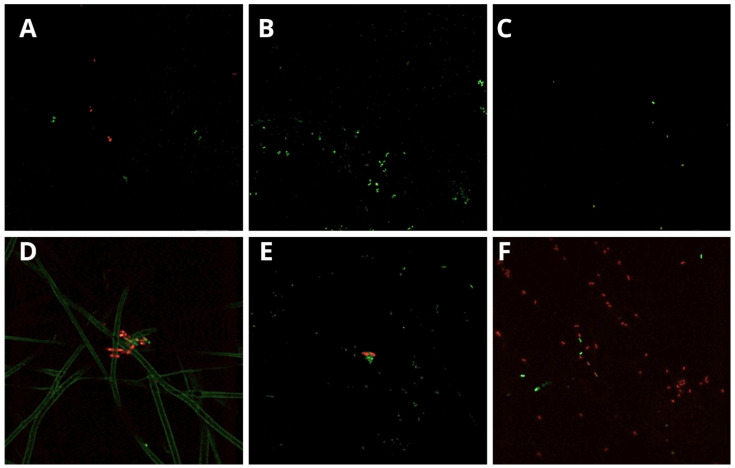
(**A**–**C**) Viable *S. mutans* and (**D**–**F**) *A. actinomycetemcomitans* cells stained in green and non-viable cells stained in red (Images obtained by Karina Cesca at the Multi-User Laboratory for Studies in Biology and Central Electron Microscopy Laboratory). (**A**) Balance between viable and non-viable *S. mutans* cells on DS membrane surface (2500× magnification). (**B**) Abundant predominance of viable *S. mutans* cells on DT membrane surface, where no non-viable cells were identified (2500× magnification). (**C**) Predominance of small proportions of viable *S. mutans* cells, with no non-viable cells identified on LP barrier (2500× magnification). (**D**) Predominance of non-viable *A. actinomycetemcomitans b* cells on DS membrane, with green traces indicating microbial mobility and foci of viable *A. actinomycetemcomitans b* cells (2500× magnification). (**E**) Greater amount of viable *A. actinomycetemcomitans b* cells in DT membrane surface, with small foci of non-viable cells (2500× magnification). (**F**) Greater amount of non-viable A. actinomycetemcomitans b cells on LP barrier (2500× magnification).

When exposed to *S. mutans*, the experimental DS group showed an equilibrium between viable and dead cells. In the DT and LP groups, the volume of viable cells was larger than that of the dead cells, and viable cells were identified in a greater proportion in the DT specimen compared to the LP (Figure 3A–C). During the inoculation with *A. actinomycetemcomitans b*, DS and LP were the groups that presented a higher proportion of dead cells. In contrast, in the DT group, viable cells were predominant (Figure 3D–F).

### 3.3. Bacterial Penetration Test

**Figure 4:**
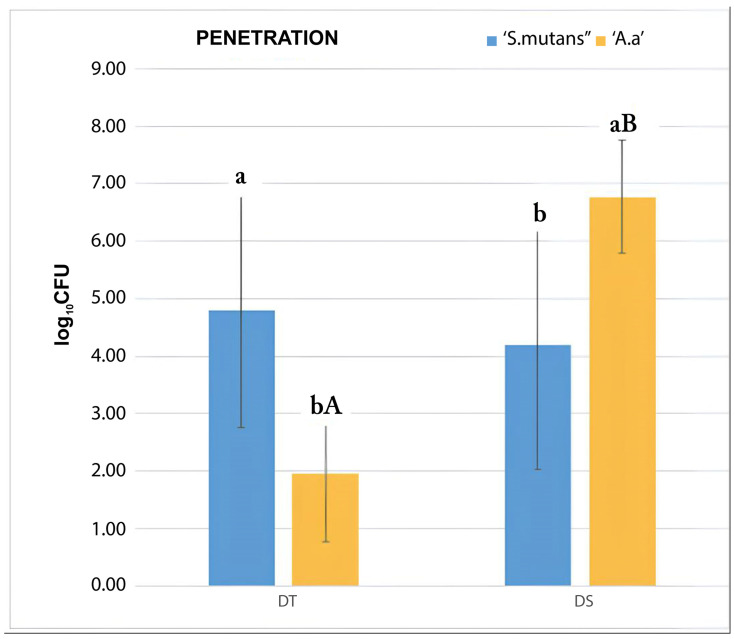
Bacterial penetration test carried out through CFU counting. Average values from the triplicate analysis. Upper-case letters indicate statistical difference between experimental groups and lower-case letters indicate statistical difference between bacterial species in the groups. DT group showed higher penetration of *S. mutans* compared to *A. actinomycetemcomitans b*, while *A. actinomycetemcomitans b* showed higher penetration in DS group compared to *S. mutans*. Penetration of *A. actinomycetemcomitans b* across DS group was higher compared to the DT and LP groups. DT and DS groups showed higher penetration of *S. mutans* compared to LP group, which prevented both bacterial species from penetration (elaborated by the authors).

The methodological apparatus completely prevented the leakage of the bacterial inoculum, which permeated completely through the membranes. The DT experimental group allowed a significantly higher amount of penetration of *S. mutans* compared to *A. actinomycetemcomitans b* (*p* = 0.009), while *A. actinomycetemcomitans b* showed a significantly higher permeability across DS membranes compared to *S. mutans’* permeability (*p* = 0.016). The penetration of *A. actinomycetemcomitans b* across DS membranes was significantly higher compared to that in the DT and LP groups (*p* < 0.01 for both). The DT and DS groups allowed a significantly higher amount of penetration of *S. mutans* compared to the LP group, which prevented both bacterial species from penetrating. The results can be visualized schematically in Table 4 and in the form of a graph in Figure 4.

## 4. Discussion

Bacterial colonization of GTR/GBR membranes and barriers directly affects the outcomes of these regenerative procedures [21,22]. Although most surgical protocols using these treatment modalities recommend a total coverage of membranes with soft tissue, the rate of premature accidental exposure reaches 45% in most cases [22]. The evidence shows a negative correlation between the exposure of biomaterials to the oral environment and the increase in clinical attachment in GBR or the osseointegration of implants associated with GBR [23,24,25,26].

Important periodontal bacteria have been found in both resorbable and non-resorbable GTR/GBR biomaterials [27,28]. Among them, attention has been drawn to the strong adhesion capacities of *A. actinomycetemcomitans* and *S. mutans* in these biomaterials [21,26,29], and therefore, they were selected to be tested in this in vitro investigation.

The bacterial species intensely colonized all of the biomaterials tested in this study, corroborating the results of other in vitro studies in which bacterial adhesion was evident in the first moments of the experimental period [22,28]. Adhesion can be explained by the structural and chemical properties of these materials, which directly influence their microbial adhesion capacity [27,30].

Synthetic resorbable membranes have the advantage of being customizable, allowing for the adjustment of their chemical and structural characteristics such as porosity, thickness, shape and others, and consequently minimizing the conditions for bacterial adhesion [31]. Still, the specimens corresponding to the synthetic biodegradable polymer membranes tested in this study allowed for a high level of adhesion of *S. mutans*, which was statistically higher than *A. actinomycetemcomitans.* A possible explanation for this fact may be based on the concept that bacterial adhesion is inversely proportional to the difference in surface free energy (SFE) between the bacteria and the substrate [32] since findings have shown that *A. actinomycetemcomitans* has a large difference from SFE relative to the surface of polymers [30]. Or even by the strong adhesion capacity of *S. mutans* to synthetic membranes mediated by glucans [26,29].

Such a strong adherent ability was also statistically significant in the collagen membrane group compared to the d-PTFE barriers. Collagen is known to be highly hydrophilic, a characteristic that grants it a great propensity for bacterial adhesion [19]. The data indicate that collagen can accelerate bacterial growth [33] and that *Streptococcus* spp. can aggregate with type I collagen molecules [34], which comprise most of the groups tested in this study.

Morphological studies regarding bacterial proliferation indicate that bacterial accumulation is conductive to retentive surfaces [33], most likely due to an increased surface availability for bacterial proliferation [35]. Despite having a hydrophobic surface with microporosities below 0.3 μm [36] and being the biomaterial with the lowest CFU count adhered to in this study, d-PTFE barriers were not protected from bacterial adhesion, agreeing with in vitro data that identified high bacterial cell counts in these biomaterials [9,22,31,37]. This information suggests that bacterial adherence, in this case, is not related to superficial porosity, and raises questions about the possibility of keeping this biomaterial exposed in the oral cavity, as has been recommended by several authors [36,38].

Although the CFU count in all of the groups tested was high, the confocal analysis did not seem to be compatible with such data, showing few isolated foci of bacterial aggregates. This can be explained by the experimental period of only two hours of bacterial incubation on the surface of the biomaterials. Thus, the bacterial adhesion stage was still initial, weak, and reversible, and non-specific physicochemical interactions may have resulted in repulsion of bacterial cells from the substrate [30] after consecutive baths in PBS and preparations of specimens with glutaraldehyde and alcohol gradients. Interestingly, in the analysis using confocal microscopy, the viability of *S. mutans* seems to have been higher than that of *A. actinomycetemcomitans* among the experimental groups. However, such information cannot be assured because of the poor bacterial adherence to the biomaterials’ surfaces and the impaired quality of the confocal microscopy images.

Our investigations prove the high capacity of bacteria to penetrate through biomaterials, including *S. mutans* and *A. actinomycetemcomitans* through collagen membranes [22,26]. Polylactic acid membranes were susceptible to the bacterial penetration of *S. mutans* after 5 h in the experimental period [39]. The results of bacterial penetration tests in the DT and DS groups corroborate those of previous studies, where bacterial penetration led to a high number of CFUs.

Statistically, the penetration of *S. mutans* was higher than that of *A. actinomycetemcomitans* in the DT membrane. At the same time, the DS group presented the inverse result, including relative to the other groups. A possible reason for this may be that the *S. mutans* cell has an approximate diameter of 0.5 to 1.0 nm, while the *A. actinomycetemcomitans* cells ranged from 1.0 to 1.5 × 0.4 at 0.5 nm [26]. In this way, *S. mutans* cells may have traversed the collagen membrane more easily. The LP specimen avoided the penetration of bacteria cells completely; however, the presence of cells on its surface in the confocal microscopy analysis confirms that such biomaterial is not prevented from bacterial adherence. One possible explanation for adherence but not penetration could be the size of the pores of the biomaterial being smaller than the size of the bacteria cell. In the DS specimens, the statistically lower adhesion of *A. actinomycetemcomitans* may justify the fact that this group experienced a significantly higher amount of penetration. This type of membrane is made by electrospinning technology and produces porosities in the membrane structure that vary in size, producing small to large pores. Thus, the bacteria’s passage through its structure may have occurred more easily.

In the LP group, bacterial penetration was not evident. The background data endorse this finding, confirming the barrier capacity of this biomaterial [9,22,40]. On the other hand, other studies have demonstrated bacterial penetration in d-PTFE membranes [28,41], even those with a high structural density and reduced porosity [37]. The non-standardization and characterization of an in vitro methodological apparatus for this type of analysis can produce false results [9,22], contributing to the results of such studies.

Given this, one of the objectives of this study was to create a reliable and efficient methodological apparatus capable of preventing biases. Thus, a three-dimensional digital print device exclusively made for the permeability test of this study, composed of a polymeric filament of PLA/ST, was developed to standardize penetration tests of in vitro biomaterials. The printed apparatus allows the test to be conducted in a practical and individualized way, and its threading and sealing system allows the adaptation of the biomaterial, ensuring its integrity and completely preventing the leakage of the bacterial inoculum, confirming the bacterial permeability only via a membrane, as the results of the penetration test demonstrate.

The choice of the 2 h experimental period is justified by the fact that a false positive result could occur in the bacterial penetration test since, after passing through the membranes, the multiplication and growth of bacteria in the BHI in the lower compartment of the apparatus could produce a CFU count that is much higher than that which actually permeated through the biomaterial. However, the utilization of a singles experimental time point may be deemed a limitation of the study, compounded by the selection of an aerobic environment, given that these conditions could potentially constrain microbial behaviors.

Alternatives for the antimicrobial incorporation and structural alteration of biomaterials used in GTR and GBR have been gaining more and more space in the literature [42,43,44] and are of great value since the consequences of bacterial contamination can make the success of regenerative treatments impossible [21]. However, the ideal antimicrobial membrane still seems far from the clinical reality given the resistance and pathogenicity of periodontal bacteria. Thus, the systematization of research on the subject must be established through the regulation of methodological in vitro devices, clinical protocols for the control of microorganisms, and the correct indication and application of biomaterials in different clinical situations.

## 5. Conclusions

Under the methodological conditions presented in this study, it can be concluded that all tested biomaterials were passive of intense bacterial adherence in different degrees, depending on the species and biomaterial characteristics. However, only the membranes allowed penetration, which was blocked by the barriers. The developed apparatus provided a complete fit to and sealing of the biomaterials, ensuring its integrity and impeding leakage of bacterial inoculum as proved by the penetration tests, thus making the standardization of in vitro penetration tests possible.

## 6. Fomentation

FGM Dental Group^®^ and Criteria^®^ provided the biomaterials used in this study. The apparatus was developed in partnership with the Management and Design Center of the Usability Laboratory (NGDLDU), linked to the graduate programs in Design and Production Engineering. The steps involving microorganisms were conducted in partnership with the Laboratory of Bacterial Molecular Genetics (GEMBAC) and microscopic visualization through the Central Laboratory of Electron Microscopy (LCME) of the Department of Biological Sciences as well as at the Center for Research in Ceramic and Composite Materials (CERMAT), of the Department of Mechanical Engineering, at the Federal University of Santa Catarina. The other materials used for the consumption, manufacture and sterilization of the apparatuses, as well as instruments for conducting the experiments, were subsidized by the researcher.

## Figures and Tables

**Table 1:** Parameters regarding the 3D printer configuration (elaborated by the authors).

3D Printer	
Layer height of the material	0.25 mm
Extruder temperature	190 °C
Heated layer temperature	34 °C

**Table 2:** Parameters regarding the printing setup of the methodological apparatus (elaborated by the authors).

Printings Setup	
Nozzle size	0.4
Bottom thickness	0.25
Filament diameter	1.75
Filament flow	100
Solid top	1.0
Solid bottom	1.0
Solid thickness of layer	0.9
Fill overlap	15
Fan activation	1.0
Fan speed	100

**Table 3:** Bacterial adherence test (CFU count) results from ANOVA two-way multivariate analysis (MANOVA) (elaborated by the authors). Average values from the triplicate analysis.

Adherence Test (MANOVA)				
**Experimental Group**	***S. mutans (β)***	***A. actinomycetemcomitans b*** **(Δ)**	**P_value_** **between Species**	**P_value_** **between Groups**
LP	5.73 (1.62)	6.08 (0.78)	**	(LP; DT) = 0.01 *β
DT	6.84 (0.21)	6.91 (0.21)	**	(DT; DS) < 0.01 *Δ
DS	6.03 (0.71)	4.98 (0.25)	0.005 *	(LP; DS) = 0.011 *Δ

* Statistically significant difference for α = 0.05; ** No significant statistical difference; *β—significant statistical difference in test with S. mutans*; Δ—*significant statistical difference in test with* com A. actinomycetemcomitans b; LP—Lumina PTFE^®^ d-PTFE barriers; DT—Lumina Coat Double Time^®^ collagen membranes; DS—Duosynt^®^ polymer membrane.

**Table 4:** Bacterial penetration test (CFU count) results from ANOVA two-way multivariate analysis (MANOVA) (elaborated by the authors). Average values from the triplicate analysis.

Penetration Test (MANOVA)				
**Experimental Group**	***S. mutans (β)***	***A. actinomycetemcomitans b*** **(Δ)**	**P_value_** **between Species**	**P_value_** **between Groups**
LP	0	0	**	(LP; DT) = 0.003 *β (LP; DS) = 0.012 *β (LP; DS) < 0.01 *Δ
DT	4.8 (2.04)	1.94 (1.19)	0.009 *	(DT;LP) = 0.03 *β *(DT; DS) < 0.01* *Δ
DS	4.19 (2.16)	6.77 (0.98)	0.016 *	(DS;LP) = 0.012 *β (DS; LP) < 0.01 *Δ (DS; DT) < 0.01 *Δ

* Statistically significant difference for α = 0.05; ** No significant statistical difference; *β—significant statistical difference in test with S. mutans*; Δ—*significant statistical difference in test with* com *A. actinomycetemcomitans b*; LP—Lumina PTFE^®^ d-PTFE barriers; DT—Lumina Coat Double Time^®^ collagen membranes; DS—Duosynt^®^ polymer membrane.

## Data Availability

The original data presented in the study are openly available in Repositório Institucional UFSC at https://repositorio.ufsc.br/handle/123456789/253538 (accessed on 26 June 2024).
